# Genetic association of the BsmI variant of vitamin D receptor gene with risk of morbid obesity

**DOI:** 10.1590/1806-9282.20231020

**Published:** 2024-07-19

**Authors:** Seyma Ozsoy, Serbulent Yigit, Ayse Feyda Nursal, Zeki Ozsoy, Mehmet Fatih Dasiran, Emin Daldal, Akin Tekcan

**Affiliations:** 1 Gaziosmanpasa University, Faculty of Medicine, Department of Physiology – Tokat, Turkey.; 2 Ondokuz Mayıs University, Faculty of Veterinary, Department of Genetics – Samsun, Turkey.; 3 Hitit University, Faculty of Medicine, Department of Medical Genetics – Çorum, Turkey.; 4 Medical Park Hospital, Department of General Surgery – Tokat, Turkey.; 5 Gaziosmanpasa University, Faculty of Medicine, Department of General Surgery – Tokat, Turkey.; 6 Samsun University, Faculty of Medicine, Department of General Surgery – Samsun, Turkey.; 7 Amasya University, Faculty of Medicine, Department of Medical Biology – Amasya, Turkey.

**Keywords:** Morbid obesity, Vitamin D receptor, BsmI, Variant

## Abstract

**OBJECTIVE:**

The aim of this study was to evaluate the vitamin D receptor (*VDR*) BsmI variant in morbidly obese patients compared with healthy normal controls.

**METHODS:**

The study included 103 patients with morbid obesity and 120 healthy individuals serving as normal controls. The DNA samples obtained from blood were genotyped using the polymerase chain reaction-restriction fragment length polymorphism (PCR-RFLP) technique. The gender, age, smoking status, triglycerides, total cholesterol, insulin, mean body mass index, and frequency of allele and genotype of the BsmI variant in the *VDR* gene in morbidly obese patients were evaluated.

**RESULTS:**

The body mass index of the patients was 47.14 ± 7.19. The *VDR* B/B, B/b, and b/b genotype frequencies were 27.2% versus 28.3%; 54.4% versus 50%; and 18.4% versus 21.7% in the morbidly obese patients and the control group, respectively. There was no statistically significant difference between patients and control subjects in the genotype and allele distribution of the *VDR* BsmI variant (p>0.05). Both patients and control genotype frequencies are consistent with Hardy-Weinberg equilibrium.

**CONCLUSION:**

The BsmI variant in the *VDR* gene may not seem to predispose to morbid obesity in our study population. Further studies with a larger number of subjects are needed to make a more precise evaluation of this relationship.

## INTRODUCTION

The escalating prevalence of obesity worldwide is a matter of significant concern^1^. The body mass index (BMI) is used to assess the severity of obesity. BMI is determined by taking a person's body weight in kilograms and dividing it by the square of their height in meters. A BMI ≥30 is defined as obesity, and a BMI value exceeding 40 is defined as morbid obesity (obesity class III)^2^. Numerous chronic diseases, including diabetes, gallstones, hypertension, nonalcoholic fatty liver disease, metabolic syndrome, and cardiovascular disorders, have been linked to obesity, according to reports^2^. The etiology of obesity is multifactorial, exhibiting a complex interplay between genes and the environment.

Vitamin D (VitD) is necessary for the homeostasis of bone tissue as well as the minerals such as calcium and phosphorus. Serum 25-hydroxyvitamin D (25(OH)D) is often used as an index of VitD nutritional status. Circulating 25(OH)D concentrations of obese individuals were found to be lower than their race, socioeconomic status, and age-matched peers^3^. Once synthesized, VitD exerts its biological effects primarily through the VitD receptor (VDR, NR1I1). VDR belongs to the nuclear hormone receptor superfamily and forms a heterodimer with the retinoid X receptor. Together, they regulate the activity of target genes containing VitD response elements^4^. VDR expression is present in all essential VitD target tissues, including the intestine, kidney, bone, and parathyroid gland. In these tissues, VDR plays a vital role in maintaining calcium and phosphorus homeostasis, ensuring proper mineral balance within the body. VDR knockout mice have been shown to have reduced body weight and are resistant to high-fat diet-induced obesity^5^. The *VDR* gene is located on chromosome 12q13 and spans 75 kb of genomic DNA, consisting of 14 exons. Within the *VDR* gene, specific single-nucleotide polymorphisms (SNPs) have been identified, one of which is the rs1544410 SNP located in the intronic region (intron 8 near the 3´ end). This SNP, also known as the BsmI variant, represents a restriction fragment length polymorphism (RFLP) of the restriction endonuclease BsmI^6^. Apart from its association with VitD levels, the BsmI polymorphism has been linked to obesity, insulin resistance, and type 2 diabetes in some study cohorts. This suggests that genetic variations in the *VDR* gene may play a role in these conditions, possibly influencing VitD metabolism and related physiological pathways^7,8^.

Based on this information, we aimed to examine whether there is a relationship between the VDR BsmI variant and morbid obesity in this study.

## METHODS

### Study population

A total of 103 morbidly obese patients (30 males and 73 females; mean age: 39.47 ± 12.60 standard deviation [SD] years) who underwent bariatric surgery in the Department of General Surgery, Gaziosmanpaşa University Research Hospital, Tokat, Turkey, were included in this study. An accurately measured BMI is obtained at the first visit to the clinic. All participants were unrelated, with no consanguinity at all, and no spouses were included. BMI was higher than 40 in all patients. Gender and age compatibility of 120 healthy volunteers (55 males and 65 females; mean age: 41.51 ± 12.11 SD years) with a normal BMI were recruited as the control group. Prior to the study, informed consent was obtained from each participant to ensure their understanding and voluntary participation. The study's protocol was reviewed and confirmed by the institutional ethics committee at Ahi Evran University (approval number: 2017-05/36). The research adhered to the principles and guidelines set forth in the Helsinki Declaration to ensure the ethical treatment of the participants and protect their rights and welfare throughout the study.

### Genotyping analysis

In this study, approximately 5 mL of peripheral blood was collected from each participant through venipuncture using Vacutainer tubes containing EDTA as an anticoagulant. DNA was removed from leukocytes using a DNA extraction kit (Sigma-Aldrich, Germany) following the manufacturer's instructions. The extracted DNA samples were stored at -20°C until analysis. To genotype the *VDR* BsmI variant, the polymerase chain reaction-RFLP (PCR-RFLP) method was employed, as previously described^9^. After PCR amplification, the products were subjected to digestion overnight at 37°C using the BsmI restriction enzyme. After the digestion process, the resulting products were separated on a 2% agarose gel, which was stained with ethidium bromide. The visualization of the separated fragments was achieved using ultraviolet transillumination. The BsmI restriction enzyme digestion yields three genotypes: B/B (825 bp), B/b (825, 650, 175 bp), and b/b (650, 175 bp).

### Statistical analysis

The data obtained from the study were analyzed using the SPSS version 22.0 software by Windows (SPSS Inc., Chicago, IL, USA). The number of sample groups in the research was determined using the G power Package program. According to the results of the analysis, the total sample size was determined to be 214 with a 95% confidence interval (CI), an alpha error rate of 0.05, and an effect size of 0.5.

To assess the statistical significance of differences between the patient and control groups, a logistic regression analysis was conducted. Odds ratios (OR) and their corresponding 95%CI were computed to quantify the associations. To compare the genotype and allele differences in the *VDR* BsmI between the patient and control groups, the chi-square test was used. In cases where sample sizes were small, Fisher's exact test was applied. The chi-square test was used to assess Hardy-Weinberg equilibrium (HWE). In all statistical analyses, two-tailed tests were used, and differences were considered statistically significant when the p-value was below 0.05 (p<0.05).

## RESULTS

In the present study, a total of 223 participants were included, comprising 103 individuals diagnosed with morbid obesity and 120 healthy adult controls. The genotyping for the *VDR* BsmI variant was performed on all subjects. Table 1 presents the characteristics of the participants in both the patient and control groups. Notably, in both study groups, there were more female participants than male participants. While there were 73 (70.9%) female and 30 (29.1%) male patients, the healthy control group consisted of 65 (54.2%) women and 55 (45.8%) men. The mean ages of morbidly obese patients and healthy individuals were 39.47 ± 12.60 and 41.51 ± 12.11, respectively. The mean BMI of patients was 47.14 ± 7.19.

**Table 1:** Baseline clinical and demographic features of the patient and control groups.

Characteristics	Control group (n = 120) (%)	Patient group (n = 103) (%)
Gender, females/males, n (%)	65/55 (54.2/45.8)	73/30 (70.9/29.1)
Age, mean ± SD, years	41.51 ± 12.11	39.47 ± 12.60
Smoking status, no/yes, n (%)		74/25 (74.7/25.3)
BMI, mean ± SD	–	47.14 ± 7.19
Triglycerides, mean ± SD, years	–	110.66 ± 107.98
Total cholesterol, mean ± SD years	–	130.33 ± 88.94
Insulin, mean ± SD years	–	26.80 ± 52.37

SD: Standard deviation; BMI: body mass index; n: number of samples.

The study results, as shown in Table 2, indicate the genotype distributions and allele frequencies of the *VDR* gene BsmI variant in all participants. However, upon analysis, no significant differences were observed between morbidly obese patients and healthy individuals concerning the *VDR* BsmI genotype and allele frequencies (p>0.05). This implies that the presence of the *VDR* BsmI variant does not appear to be associated with morbid obesity in this study population. Both patients and control genotype frequencies are consistent with HWE (p>0.05).

**Table 2:** Genotype and allele frequencies of the *VDR BsmI* gene variant in the patient and control groups.

VDR *BsmI*		Patient group n = 103 (%)	Control group n = 120 (%)	X^2^	p-value	OR (95%CI)
Genotypes						
	B/B	28 (27.2)	34 (28.3)	1.62	>0.05	0.855 (0.40–1.78)
	B/b	56 (54.4)	60 (50.0)		>0.05	0.864 (0.50–1.47)
	b/b	19 (18.4)	26 (21.7)		>0.05	0.854 (0.35–2.04)
	B/B+B/b:b/b	84:19	94:26		>0.05	1.222 (0.63–2.39)
	B/B:B/b+b/b	28:75	34:86		>0.05	0.944 (0.52–1.70)
	HWE	0.33	0.96			
Alleles						
	B	112 (19.6)	128 (24)		>0.05	1.042 (0.71–1.51)
	b	94 (80.3)	112 (76)			

n: number of samples; p-value: the statistical significance; HWE: Hardy-Weinberg equilibrium.

## DISCUSSION

Obesity has emerged as a pressing global health concern, affecting a growing number of countries due to its high prevalence, substantial economic costs, and significant health implications^2^. The excess of macronutrients in fatty tissues stimulates the release of inflammatory mediators such as TNFα and IL-6^10^. VitD deficiency is positively associated with serum levels of inflammatory markers such as IL-6, TNFα, and C-reactive protein in obese individuals^11^. VitD may have immunomodulatory effects with its anti-adipogenic properties. This may help reduce inflammation in fatty tissues^12^. It has been reported that there is a relationship between VitD deficiency during pregnancy and gestational diabetes^13^.

Metabolic regulation of VitD in adipose tissue is dependent on VDR. VDR belongs to the steroid hormone receptor superfamily, and it has been found to play a significant role in the development of obesity. Data show a widespread expression of VitD-related metabolic enzymes and VDR in human adipose tissue^14^. Since VDRs are present in various body tissues, their gene polymorphisms may influence the risk of VitD-related metabolic disorders and regulate the receptor's effectiveness based on VitD status^15^.

Genetic changes in the *VDR* gene can cause defects in gene activation. But they also support cell proliferation, differentiation, calcium metabolism, immune function, etc^16^. Among the various polymorphisms identified in the *VDR* gene to date, BsmI, Apal, TaqI, and FokI have been studied the most^17^. Various *VDR* polymorphisms have been associated with type 2 diabetes mellitus, insulin release^18^, and metabolic parameters related to obesity^19^. The *VDR* BsmI variant is believed to affect the gene's mRNA stability and gene transcription, potentially altering the expression of other genes. Studies conducted on different populations have reported varying associations between the BsmI variant and obesity. In a study conducted in France, it was shown that the *VDR* BsmI b/b genotype is predisposed to obesity compared to B/B and B/b genotypes^20^. Another study reported an association between body weight and BsmI variant in men in age-adjusted analysis^21^. In the study conducted with Saudi men, it was found that the BsmI b/b genotype was higher in the obese group compared to the lean group^22^. Additionally, those with the b/b genotype had higher BMI and HOMA-IR than those with the B/B and B/b genotypes. A study in obese children in Turkey found that BsmI polymorphism had a positive effect on the formation of obesity, metabolic syndrome, and hepatosteatosis^23^. However, some studies have not observed significant relationships between the *VDR* BsmI variant and obesity-related parameters. For example, a Polish cohort of postmenopausal women did not find any association of the *VDR* BsmI variant with BMI, total fat volume, or visceral fat^24^. Similarly, a study on obese and nonobese women with polycystic ovary syndrome did not show a significant association with the *VDR* BsmI variant^25^.

In the current study, we investigated the genetic association between the *VDR* BsmI variant and morbid obesity. The genotype and allele frequencies of this variant showed no statistically significant difference between the morbidly obese patients and the healthy controls (Table 2). These findings were consistent with some previous studies.

This study has some limitations. The first limitation is that it evaluates only one variant of the *VDR* gene. Other variants of this gene may also affect the development of morbid obesity. Additionally, gene–environment and gene–gene interactions were not evaluated. The final limitation is that the VDR blood level was not assessed. However, the strength of our study is that only patients from a single region were included.

## CONCLUSION

Identifying the relationship between genetic variants and obesity is crucial to understanding pathogenesis. This study represents a valuable step in investigating the potential link between *VDR* BsmI and obesity susceptibility. Large samples and replication in different ethnic groups are required to evaluate the results. The results may help develop personalized therapeutic approaches.
